# Supplementary material for: Group II intron inhibits conjugative relaxase expression in bacteria by mRNA targeting
Source: eLife. 2018 Jun 15;7:e34268. doi: 10.7554/eLife.34268 (PMC6003770; doi:10.7554/eLife.34268)
Supplement: Supplementary file 1. — Supplementary Table 1: Plasmids. Describes plasmids used in this study. Supplementary Table 2: DNA oligonucleotides. Describes DNA oligonucleotides used in this study. Supplementary Table 3: Primers used in qRT-PCR. Describes the primers used for qRT-PCR. [file elife-34268-supp1.docx]

**Title: Group II intron inhibits conjugative relaxase expression in bacteria by mRNA targeting**

**Running: Group II intron targets mRNA**

**Authors:**

Guosheng Qu^1^, Carol Lyn Piazza^1^, Dorie Smith^1^, Marlene Belfort^1,2^

**Supplementary Information:**

**Tables**

**Supplementary Table 1. Plasmids**

| Name | Description | Reference |
| --- | --- | --- |
| pCY20 LtrB | Nisin inducible pCY20 (*spec^R^*) for expression of wild-type LtrB and for LtrB mRNA related mutageneses, Int^-^ | ([Novikova et al., 2014](#_ENREF_7)) |
| pCY20 LtrB::Ll.LtrB | Nisin inducible pCY20 (*spec^R^*) for expression of wild-type LtrB interrupted by wild-type full-length Ll.LtrB intron, and for LtrB and intron related mutageneses, Int^+^ | Belfort lab |
| pCY20 LtrB IBS (mRNA_IBSm) | IBS region (CAC ATC CAT AAC CA) of LtrB CDS on pCY20 LtrB mutated to: GTG TAC GTG TTG GT | This work |
| pCY20 LtrB IBSm* (mRNA_IBSm*) | IBS region (CAC ATC CAT AAC CA) of LtrB CDS on pCY20 LtrB mutated to: CAC ATC GTG TTG GT to complement the Intron_EBSm* mutant. | This work |
| pCY20 LtrB::Ll.LtrB Triad | The catalytic triad AGC of the Ll.LtrB intron on pCY20 LtrB::Ll.LtrB mutated to GAU | This work |
| pLNRK smEx | Nisin inducible pLNRK (*cam^R^*) for expression of small ligated LtrB exons- 15nt of 5′ exon with 3nt of 3′ exon | Belfort lab |
| pLNRK smEx::Ll.LtrB | Nisin inducible pLNRK (*cam^R^*) for expression of wild-type full-length Ll.LtrB intron flanked by small exons (15+3 nt as pLNRK smEx), and for Ll.LtrB intron related mutageneses | Belfort lab |
| pLNRK smEx-nLIC | Nisin inducible pLNRK (*cam^R^*) for co-expression of small ligated LtrB exons and downstream LtrA tagged with Intein-Chitin Binding Domain | Belfort lab |
| pGpII(SA)-CUP1(6XMS2) | Yeast vector for constitutive expression of streptavidin aptamer inserted, ORF deleted intron, that is flanked by small exons and followed by yeast CUP1 coding sequence and 6X MS2 | ([Qu et al., 2014](#_ENREF_8)) |
| pLNRK smEx::Ll.LtrB ΔORF (SA)-nLIC | Nisin inducible pLNRK (*cam^R^*) for expression of streptavidin aptamer inserted, ORF deleted intron, flanked by small exons. Downstream LtrA tagged with Intein-Chitin Binding Domain as in pLNRK smEx-nLIC | This work |
| pLNRK smEx::Ll.LtrB ΔORF-nLIC | Nisin inducible pLNRK (*cam^R^*) for expression of ORF deleted intron, flanked by small exons. Downstream LtrA tagged with Intein-Chitin Binding Domain | ([Qu et al., 2016](#_ENREF_9)) |
| pLNRK smEx::Ll.LtrB RT | The LtrA RT catalytic resisues YADD encoded by the Ll.LtrB intron on pLNRK smEx::Ll.LtrB mutated to YAAA | This work |
| pLNRK smEx::Ll.LtrB EN | The LtrA EN catalytic residues EIHHV encoded by the Ll.LtrB intron on pLNRK smEx::Ll.LtrB mutated to AIAAA | This work |
| pLNRK smEx::Ll.LtrB EBSm  (Intron_EBSm) | The δ-EBS1 region (TGG TTG TG) of the Ll.LtrB intron on pLNRK smEx::Ll.LtrB mutated to: ACC AAT AC | This work |
| pLNRK smEx::Ll.LtrB EBSm* (Intron_EBSm*) | The δ-EBS1 region (TGG TTG TG) of the Ll.LtrB intron on pLNRK smEx::Ll.LtrB mutated to: ACC AAT AC to complement the mRNA_IBSm* mutant. IBS1 region (CAT AAC) was mutated to: GTG TTG, and δ’ region (CA) mutated to :GT to enable splicing of the intron. | This work |
| pDL278 LtrB | pDL278 (*spec^R^*) plasmid for expression of wild-type LtrB that is driven by p23 constitutive promoter | This work |
| pDL278 LtrB::Ll.LtrB | pDL278 (*spec^R^*) plasmid for expression of wild-type LtrB interrupted by wild-type full-length Ll.LtrB intron that is driven by p23 constitutive promoter | This work |
| pAMJ328 LtrB | P170 (*erm^R^*) plasmid for expression of wild-type LtrB that is driven by a pH inducible promoter | This work |
| pAMJ328 LtrB::Ll.LtrB | P170 (*erm^R^*) plasmid for expression of wild-type LtrB interrupted by wild-type full-length Ll.LtrB intron that is driven by a pH inducible promoter | This work |
| pET11a-HS-GFP(EcI5) | pET11a (*amp^R^*) for expression of EcI5 intron homing-site sequence fused to GFP(S65T) that is driven by T7 IPTG inducible promoter | This work |
| pET11a-GPII-GFP(EcI5) | pET11a (*amp^R^*) for expression of homing-site sequence interrupted by EcI5 intron and fused to GFP(S65T) that is driven by T7 IPTG inducible promoter | This work |
| pET11a-HS-GFP(BhI1) | pET11a (amp^R^) for expression of BhI1 intron homing-site sequence fused to GFP(S65T) that is driven by T7 IPTG inducible promoter | This work |
| pET11a-GPII-GFP(BhI1) | pET11a (amp^R^) for expression of homing-site sequence interrupted by BhI1 intron and fused to GFP(S65T) that is driven by T7 IPTG inducible promoter | This work |

**Supplementary Table 2. DNA oligonucleotides**

| IDT No. | Sequence (5′-3′) | Description |
| --- | --- | --- |
| Northern blotting | | |
| 4685 | AAAAATGATATGGTTATGGATGTG | LtrB mRNA exon-exon splice junction probe |
| 1073 | GTACCTTAAACTACTTGACTTAACACC | Ll.LtrB intron probe (also used in primer extensions) |
| 5374 | GACCAAGCACCGCATTATTT | LtrB 5′ exon probe |
| 5012 | CATCATAAGCTTTGCCGCTTT | LtrB 3′ exon probe |
| 4972 | AAAATACATACGATTCGAGCACCA | EcI5 mRNA probe |
| 4970 | CAATTATTCAGGCGACTTCATGTCG | EcI5 intron probe |
| 4975 | CCCTTTTTTCATACTAAAAAAGCACA | BhI1 mRNA probe |
| 4973 | TAGAGAATATGCGCATGCCTGG | BhI1 intron probe |
| 0861 | CGAGCTGACGACAACCATGCACCACC | *L. lactis* 16S rRNA probe |
| RT primer extension | | |
| 4836 | AACTACTTGACTTAACACCCTATCT | Ll.LtrB intron primer |
| 4916 | TTACTTGAATTTCAGAGAGCTTGCT | LtrB 5′ exon primer |
| 5078 | GCGGCCGCAGAATTAAAAATG | 3′ exon of small mRNA specific-for RNA pull down |
| 5127 | TATTACGCTACCGAATGTCTCATG | *L. lactis* 6S non-coding RNA primer |
| Construction of plasmids for RNA pull-down | | |
| 4942 | CTC GAG TCT AGA GAA CAC ATC CAT AAC GTG CGC CCA | SA-aptamer-containing ORF-free LtrB intron PCR primer-forward (XhoI) |
| 4943 | GCGGCCGCAGAA TTA AAA ATG ATA TGG TGA AG | SA-aptamer-containing ORF-free LtrB intron PCR primer-reverse (NotI) |
| 5051 | TCGAGTCTAGAGAACACATCCATAACCATATCATTTTTAATTCTGC | Ligated exons in SA-containing intron-forward |
| 5052 | GGCCGCAGAATTAAAAATGATATGGTTATGGATGTGTTCTCTAGAC | Ligated exons in SA-containing intron-reverse |
| pDL278 Cloning | | |
| 4768 | GGATCCTAACTCAGCAGCTCTCTGAAATTC | LtrB 5′ exon PCR primer with (BamHI) |
| 4767 | GCATGCTTATAGTATTTTTCCTTTATTTTC | LtrB 3′ exon PCR primer with (SphI) |
| pAMJ328 Cloning | | |
| 4766 | ACTAGTTAACTCAGCAGCTCTCTGAAATTC | LtrB 5′ exon PCR primer with SpeI site |
| 4769 | CTGCAGTTATAGTATTTTTCCTTTATTTTC | LtrB 3′ exon PCR primer with PstI site |
| EcI5/BhI1 Cloning | | |
| 4831 | AAGCTTATGAAACTGGTGCTCGAATCGTATGTATTTTTCTGGAAA GGAGAAGAACTTTTCACTG | HS_EcI5 (HindIII) PCR primer forward |
| 4832 | AAGCTTATGAAACTGGTGCTCGAATGTGCGA | GPII_EcI5_GFP SOEing: GpII (HindIII) forward |
| 4833 | CTTCTCCTTTCCAGA AAA ATACATACGATCGG | GPII_EcI5_GFP SOEing: GPII reverse |
| 4834 | ATTTTTCTGGAA AGGAGA AGA ACTTTTCACTG | GPII_EcI5_GFP SOEing: GFP forward |
| 4827 | ACTAGTTTTGTATAGTTCATCCATGCCATGTG | GFP (SpeI) PCR primer reverse |
| 4826 | AAGCTTATGAAGCACAATCCAATCGGGTTGTGCTTTTTTAGTATGAAAAAAGGGCGCTGGAAAGGAGAAGAACTTTTCACTG | HS_BhI1 (HindIII) PCR primer forward |
| 4828 | AAGCTTATGAAGCACAATCCAATCGGGTTGTGCTTTTTTAG | GPII_BhI1_GFP SOEing: GpII (HindIII) forward |
| 4829 | CTTCTCCTTTCCAGCGCCCTTTTTTCATAATCGAG | GPII_BhI1_GFP SOEing: GPII reverse |
| 4830 | AGGGCGCTGGAAAGGAGAAGAACTTTTCACTG | GPII_BhI1_GFP SOEing: GFP forward |
| 4824 | TATGAAGCTTGGTACCACGCGTGAGCTCGTCGACAGTACTACTAGTG | PET11a linker forward (NdeI,BamHI) |
| 4825 | GATCCACTAGTAGTACTGTCGACGAGCTCACGCGTGGTACCAAGCTTCA | PET11a linker reverse (NdeI,BamHI) |
| Sequencing of LtrB and Intron Mutants | | |
| 0341 | AAGGGACGCGTGCCACGTTGTGTCTCAAAATCTCTG | pCY20 specific |
| 4761 | CGTTACACTCATTGGACTCATC | Intron specific |
| 4762 | GTATGGCTATGCCCGGAATAC | Intron specific |
| 4763 | GTGCGCCCAGATAGGGTGTTAAGTCAAGTAG | Intron specific |
| 4764 | GGG ATA TGA TAT ACG AGT AAG GAG | Intron specific |
| 4765 | AGTCTCCAGGTAACTCTCAC | LtrB 3′ exon specific |
| 4842 | TCTTTAAAAGACGGAACTTACTATCCT | Intron specific |
| 5043 | GCTGAACACGGCGTTACTGAAGGGACTC | LtrB 3′ exon specific |
| 5044 | GCGGTGCTTGGTCATCACCTCATCC | LtrB 5′ exon specific |
| Site-Directed Mutagenesis | | |
| 4888 | CACGTCGATCGTGAAGTGTACGTGTTGGTTATCATTTTT AATTC | LtrB IBS mutation (mRNA_IBSm)-Forward-with pCY20 LtrB template |
| 4889 | GAATTAAAAATGATAACCAACACGTACACTTCACGATCG ACGTG | LtrB IBS mutation (mRNA_IBSm)-Reverse- with pCY20 LtrB template |
| 6079 | ATAAAGATTCGTAGAATTAAAAATGATAACCAACACGATGTGTTCACGATCGACGTGGG | LtrB_IBSm* (mRNA_IBSm*)-Reverse-with pCY20 LtrB template |
| 6080 | CCCACGTCGATCGTGAACACATCGTGTTGGTTATCATTTTTAATTCTACGAATCTTTAT | LtrB_IBSm* (mRNA_IBSm*)-Forward-with pCY20 LtrB template |
| 4891 | AAGAAAGGTAAGTTAACCAATACGACTTATCTGTTATCA | Ll.LtrB intron EBS1 mutation (Intron_EBSm)-Forward |
| 4892 | TGATAACAGATAAGTCGTATTGGTTAACTTACCTTTCTT | Ll.LtrB intron EBS1 mutation (Intron_EBSm)-Reverse |
| 6081 | GTACCACTAGTTCTAGAGAACACATCGTGTTGGTGCGCCCAGATAGGGTGTTAAGTCA | Ll.LtrB intron EBSm* (Intron_EBSm*)-PCR1-Forward-with pLNRKsmEx::Ll.LtrB(EBS) template |
| 6082 | TGACTTAACACCCTATCTGGGCGCACCAACACGATGTGTTCTCTAGAACTAGTGGTAC | Ll.LtrB intron EBSm* (Intron_EBSm*)-PCR1-Reverse-with pLNRKsmEx::Ll.LtrB(EBS) template |
| 6083 | AGCTTGAGCTCTCTAGAAACGTGAAGTAGGGAGGTACC | Ll.LtrB intron EBSm* (Intron_EBSm*)-PCR2-Forward-with PCR1 plasmid product template |
| 6084 | GGTACCTCCCTACTTCACGTTTCTAGAGAGCTCAAGCT | Ll.LtrB intron EBSm* (Intron_EBSm*)-PCR2-Reverse-with PCR1 plasmid product template |
| 4359 | CGAACGAACAATAACAGGATCGTATACTCCGAGAGGGGT ACG | Ll.LtrB intron Triad mutation-Forward |
| 4360 | CGTACCCCTCTCGGAGTATACGATCCTGTTATTGTTCGT TCG | Ll.LtrB intron Triad mutation-Reverse |
| 4940 | GAAATACGTCCGGTATGCGGCAGCATTCATTATCTCTGT TAAAG | Ll.LtrB intron RT domain mutation-Forward |
| 4941 | CTTTAACAGAGATAATGAATGCTGCCGCATACCGGACGT ATTTC | Ll.LtrB intron RT domain mutation-Reverse |
| 4938 | GAAAATACTTCCTATGCAATTGCAGCAGCAAATAAGGTC AAAAAT | Ll.LtrB intron EN domain mutation-Forward |
| 4939 | ATTTTTGACCTTATTTGCTGCTGCAATTGCATAGGAAGT ATTTTC | Ll.LtrB intron EN domain mutation-Reverse |
| 5′and 3′ RACE | | |
| 6070 | GATAAGTGGTCCACTTGCCA | LtrB 3′ exon Gene Specific Primer for 5′ RACE |
| 6074 | GCAACTTGTTTCTGGTCATGGT | LtrB 5′ exon Gene Specific Primer for 3′ RACE |

**Supplementary Table 3. Primers used in qRT-PCR**

| IDT No. | Sequence (5'-3') | Description | Amplicon Length (bp) | Primer pair Amplification  Efficiency (%) |
| --- | --- | --- | --- | --- |
| 5003 | CTCAAGAAGTTGGAGGGTGAA | Ll.LtrB intron primer, for Pre-mRNA and Intron targets-Forward | 138 | 84.2 (Int^+^) |
| 5004 | AATGAAGTCGTCCGCATACC | Ll.LtrB intron primer, for Pre-mRNA and Intron targets -Reverse |  |  |
| 5005 | TGCTTGGTCATCACCTCATC | LtrB 5′ exon-intron junction primer, for 5′ ex-Intron target - Forward | 168 | 106.2 (Int^+^) |
| 5006 | TATCTGGGCGCACGTTATG | Ll.LtrB intron primer, for 5′ ex-Intron target - Reverse |  |  |
| 5007 | CCTCCCTACTTCACCATATC | Ll.LtrB intron - 3′ exon junction primer, for Intron-3′ ex target -Forward | 200 | 118.2 (Int^+^) |
| 5008 | GTGAGAGTTACCTGGAGAC | Intron- 3′ exon primer, for Intron-3′ ex target -Reverse |  |  |
| 5011 | ATCGTGAACACATCCATAACCA | LtrB mRNA splice junction primer, for mRNA target-Forward | 117 | 101.6 (Int^-^) 111.7 (Int^+^) |
| 5012 | CATCATAAGCTTTGCCGCTTT | LtrB mRNA 3′ exon primer, for mRNA target -Reverse |  |  |
| 4978 | CGGAATCGACTGATTTAGCG | dm9 ([Magnani et al., 2008](#_ENREF_6)); CopA primer-Forward | 148 | 102.9 (Int^-^) 104.4 ( Int^+^) |
| 4979 | AACAGGTAAGGCTTCACCTG | dm10 ([Magnani et al., 2008](#_ENREF_6)); CopA primer-Reverse |  |  |
| 5207 | GCATCACCACGCATTACAAG | LtrB 5′ exon primer, for 5′ transcription targets -Forward | 114 | 98.9 (Int^-^)  102.5 (Int^+^) |
| 5208 | ATTTCAGAGAGCTGCTGAGTTA | LtrB 5′ exon primer, for 5′ transcription targets -Reverse |  |  |
